# Neural responses to biological motion distinguish autistic and schizotypal traits

**DOI:** 10.1093/scan/nsad011

**Published:** 2023-02-27

**Authors:** Matthew Hudson, Severi Santavirta, Vesa Putkinen, Kerttu Seppälä, Lihua Sun, Tomi Karjalainen, Henry K Karlsson, Jussi Hirvonen, Lauri Nummenmaa

**Affiliations:** Turku PET Centre, University of Turku, Turku 20520, Finland; Turku University Hospital, Turku 20520, Finland; School of Psychology, University of Plymouth, Plymouth PL4 8AA, UK; Brain Research & Imaging Centre, Faculty of Health, University of Plymouth, Research Way, Plymouth PL6 8BU, UK; Turku PET Centre, University of Turku, Turku 20520, Finland; Turku University Hospital, Turku 20520, Finland; Turku PET Centre, University of Turku, Turku 20520, Finland; Turku University Hospital, Turku 20520, Finland; Turku PET Centre, University of Turku, Turku 20520, Finland; Turku University Hospital, Turku 20520, Finland; Department of Medical Physics, Turku University Hospital, Turku 20520, Finland; Turku PET Centre, University of Turku, Turku 20520, Finland; Turku University Hospital, Turku 20520, Finland; Department of Nuclear Medicine, Huashan Hospital, Fudan University, Shanghai 200040, China; Turku PET Centre, University of Turku, Turku 20520, Finland; Turku University Hospital, Turku 20520, Finland; Turku PET Centre, University of Turku, Turku 20520, Finland; Turku University Hospital, Turku 20520, Finland; Department of Radiology, University of Turku and Turku University Hospital, Turku 20520, Finland; Medical Imaging Centre, Department of Radiology, Tampere University and Tampere University Hospital, Tampere 33100, Finland; Turku PET Centre, University of Turku, Turku 20520, Finland; Department of Psychology, University of Turku, Turku 20520, Finland

**Keywords:** action observation network, neural synchronization, social perception, naturalistic fMRI, social neuroscience

## Abstract

Difficulties in social interactions characterize both autism and schizophrenia and are correlated in the neurotypical population. It is unknown whether this represents a shared etiology or superficial phenotypic overlap. Both conditions exhibit atypical neural activity in response to the perception of social stimuli and decreased neural synchronization between individuals. This study investigated if neural activity and neural synchronization associated with biological motion perception are differentially associated with autistic and schizotypal traits in the neurotypical population. Participants viewed naturalistic social interactions while hemodynamic brain activity was measured with fMRI, which was modeled against a continuous measure of the extent of biological motion. General linear model analysis revealed that biological motion perception was associated with neural activity across the action observation network. However, intersubject phase synchronization analysis revealed neural activity to be synchronized between individuals in occipital and parietal areas but desynchronized in temporal and frontal regions. Autistic traits were associated with decreased neural activity (precuneus and middle cingulate gyrus), and schizotypal traits were associated with decreased neural synchronization (middle and inferior frontal gyri). Biological motion perception elicits divergent patterns of neural activity and synchronization, which dissociate autistic and schizotypal traits in the general population, suggesting that they originate from different neural mechanisms.

The disruption of social processes is evident across numerous neurodevelopmental, mental health and neurodegenerative conditions (Cotter *et al.*, 2018; Porcelli *et al.*, 2019). Social behavior is considered a transdiagnostic domain characterized along a continuum of functionality encompassing affiliative, emotional, perceptual and cognitive processes relating to self and others (Kennedy and Adolphs, 2012; Uljarević *et al.*, 2020; Förstner *et al.*, 2022) and the neural mechanisms that underpin them (Schilbach *et al.*, 2016; Barlati *et al.*, 2020; Lobo *et al.*, 2023).

Both autism and schizophrenia are characterized by difficulties in social interactions (American Psychiatric Association, 2013), which are associated with atypical perceptual and cognitive processes in response to social stimuli (King and Lord, 2011; Sasson *et al.*, 2011; Abdi and Sharma, 2004; Hommer and Swedo, 2015; Pina-Camacho *et al.*, 2016). While these conditions can be distinguished by other core features (e.g. restricted interests and behaviors in autism and delusions and hallucinations in psychosis), the phenotypic convergence of social features contributes to uncertain identification (Nylander, 2014), mutual co-occurrence (Barneveld *et al.*, 2011; Kincaid *et al.*, 2017; Zheng *et al.*, 2018; De Crescenzo *et al.*, 2019; Lugo-Marína *et al.*, 2019; Kiyono *et al.*, 2020) and heritability estimates (Sullivan *et al.*, 2012; Wieckowski *et al.*, 2017). The degree of autistic and schizotypal traits varies in the general population (Landry and Chouinard, 2016; van Os and Reininghaus, 2016), and these dimensions are correlated, especially with respect to social behavior (Zhou *et al.*, 2019; Isvoranu *et al.*, 2021). However, it is unclear whether this convergence reflects a shared etiology or dissociable etiologies that manifest in overlapping phenotypes (DeVylder and Oh, 2014; Chisholm *et al.*, 2015). Difficulties in interpreting the agency, intentionality, emotion and purpose of other’s behavior (Couture *et al.*, 2010; Pinkham *et al.*, 2019) may be the precipitating feature that makes complex social interactions more challenging (Froese *et al.*, 2013; Gallagher and Varga, 2015). Social perceptual processes may therefore provide the origin for the high-level social difficulties experienced and potentially present a point of divergence between the autistic and schizophrenic neurophenotypes. A key issue is, therefore, to identify neurocognitive correlates of social behavior that may discriminate between these traits.

The distinctive kinematic profile of biological motion, and the social cues inherent in facial and bodily movements, allows one to not only detect intentional agents but spontaneously and implicitly infer the hidden mental states that are driving their behavior. The visual perception of social stimuli reliably elicits widespread neural activity in a distributed and hierarchical network of cortical regions, from category-specific regions of early visual areas to motion-processing regions sensitive to the intentionality of other actions and visuomotor areas of the parietal and prefrontal cortices (Decety and Grèzes, 1999; Caspers *et al.*, 2010; Grosbras *et al.*, 2012; Pavlova, 2012). The neural activity is linearly related to the frequency and prominence of facial/bodily motion that can be seen (Bartels and Zeki, 2004; Lahnakoski *et al.*, 2012). Furthermore, the neural activity becomes synchronized between individuals in many of these areas during the perception of complex social interactions (Hasson *et al.*, 2004; Bolt *et al.*, 2018). Neural synchronization reflects the extent to which voxel-specific neural activity correlates between individuals. A high degree of synchronization suggests a population-wide similarity in the neural response to a common stimulus and may provide the basis by which we share an understanding of the world with other people that is necessary for many key high-level social functions (Zaki and Ochsner, 2009; Adolphs *et al.*, 2016; Hasson and Frith, 2016; Nummenmaa *et al.*, 2018; Hudson *et al.*, 2020; Redcay and Moraczewskib, 2020). A low degree of synchronization reflects a variable neural response that suggests a more idiosyncratic perception or interpretation of a stimulus that is particular to that individual. However, it is unknown whether the synchronization of neural activity correlates with the extent and intensity of biological motion being observed and how this may differ from the magnitude of activity typically assessed as a neural correlate of social perception.

Differential patterns of neural magnitude and synchronization in response to social stimuli may provide important insights into individual differences in social behaviors that are associated with autism and schizophrenia. Although individuals with either condition detect biological motion and discriminate different actions, such as dancing *vs* fighting (Cusack *et al.*, 2015) and walking direction (Keane *et al.*, 2018), they show a difficulty in making higher-level inferences regarding the emotional and intentional dispositions of people depicted (Corrigan, 1997; Kaiser and Shiffrar, 2009; Hudson *et al.*, 2012a, 2021; Savla *et al.*, 2013; Okruszek and Pilecka, 2017; Todorova *et al.*, 2019). Moreover, these difficulties correlate with autistic and schizotypal traits in the general population (Gray *et al.*, 2011; Hudson *et al.*, 2012b; Blain *et al.*, 2017). Those with autism and schizophrenia consistently exhibit atypical neural activity in the network of regions implicated in social perception (Sugranyes *et al.*, 2011; Philip *et al.*, 2012; Mehta *et al.*, 2014; Glerean *et al.*, 2016; Yang and Hofmann, 2016; Jáni and Kašpárek, 2017; Barlati *et al.*, 2020; Chan and Han, 2020), and descriptive comparisons between those with autism and schizophrenia have revealed comparably atypical neural activity when perceiving a range of social stimuli (for reviews, see Abdi and Sharma, 2004; King and Lord, 2011; Sasson *et al.*, 2011). Direct comparisons between autistic and schizophrenic groups suggest quantitative, rather than qualitative, differences in neural activity in key brain regions involved in the perception of and reasoning about other people. Autistic groups exhibit a larger reduction in activity than schizophrenic groups in the prefrontal cortex, temporal parietal junction, amygdala and cingulate cortex but increased activity in the superior temporal sulcus (Pinkham *et al.*, 2008; Sugranyes *et al.*, 2011; Eack *et al.*, 2017). Neural activity associated with biological motion perception is positively correlated with both autistic (Thurman *et al.*, 2016; Puglia and Morris, 2017) and schizotypal traits (Platek *et al.*, 2005; Hur *et al.*, 2016). Furthermore, there is a decreased neural synchronization with other people in both autistic (Hasson *et al.*, 2009; Salmi *et al.*, 2009) and schizophrenic groups (Lerner *et al.*, 2018; Mäntylä *et al.*, 2018) that may imply a more idiosyncratic and variable perception and interpretation of the world, which may contribute to a difficulty in establishing shared perspectives with other people.

## The current study

No studies have assessed neural synchronization in response to the perception of social stimuli and how this is related to atypical social behavior associated with autistic and schizophrenic traits. The aims of this study are 2-fold: first, to establish how the extent of neural synchronization between individuals during the perception of biological motion converges or diverges from the magnitude of neural activity that traditionally defines the action observation network. Importantly, the degree of synchronization is theoretically independent of the amplitude of the neural response, even if they are both associated with the same stimulus features (Nastase *et al.*, 2019). An increase in synchronization would suggest a stimulus-specific neural response that is shared across individuals, whereas a decrease in synchronization would suggest a stimulus-specific neural response that is distinct between individuals. Furthermore, measures of neural synchrony can reveal regions of neural response that general linear model (GLM) approaches do not show, where synchronization varies with the stimulus, but the overall neural amplitude does not vary (Hejnar *et al.*, 2007; Pajula *et al.*, 2012; Xu *et al.*, 2020). There is, therefore, a compelling reason to establish how both the amplitude and reliability of neural activity vary to reveal regions that are implicated in biological motion perception with either highly generic or individual neural profiles. To this end, we had participants who viewed a series of movie clips of complex and dynamic naturalistic social interactions in a magnetic resonance imaging (MRI) scanner. These clips were observed by a separate sample of participants who gave a continuous rating of the extent of biological motion present, which provided a behavioral measure that was correlated with both the magnitude and synchronization of neural activity in each voxel.

The second aim was to investigate how differential patterns of neural magnitude and synchronization associated with biological motion perception may differentiate the overlapping social difficulties characterized by autistic and schizotypal traits in the general population. Each participant completed the Autistic Spectrum Quotient (AQ: Baron-Cohen *et al.*, 2001) and Oxford–Liverpool Inventory of Feelings and Experiences (O-LIFE: Mason *et al.*, 2005) to measure autistic and schizotypal traits, respectively. Previous research has shown that the AQ and O-LIFE correlate positively in the general population, especially with respect to the social skills and introvertive anhedonia subscales that measure social behavior (Russell-Smith *et al.*, 2011). We were, therefore, specifically interested not only in the overall scores but also in the subscales indicative of social behavior—the social skills subscale of the AQ and the introversion subscale of the O-LIFE—and the extent to which they were associated with individual differences in the magnitude of neural activity and neural synchronization in response to biological motion perception.

## Method

### Participants

Participants (*N* = 104) were recruited from the University of Turku and the wider community. After exclusions (two due to scanning artifacts, two due to gross brain abnormalities and three due to incomplete questionnaire data), 97 participants were included in the analysis (48 females, mean age = 31.3 years, s.d. = 9.4 and 56% with higher education qualification). All participants were screened using standard MRI exclusion criteria, in addition to neurological, neuropsychiatric and psychotropic substance use–related contraindications. Participants gave written informed consent prior to the study and were paid for participation. The study was approved by the ethics committee of the Hospital District of South West Finland in accordance with the Declaration of Helsinki.

## Materials and stimuli

### Questionnaires

Participants completed the questionnaires online after the scanning session. The AQ (Baron-Cohen *et al.*, 2001) is a 50-item self-report questionnaire that measures autistic traits in the general population and contains five subscales relating to social skills, attention to detail, attention switching, imagination and communication. Responses are made on a 4-point scale (definitely/slightly agree and definitely/slightly disagree) and scored as 1 or 0 (26 reverse-scored items), with higher scores indicating greater autistic traits. The O-LIFE (Mason *et al.*, 2005) is a 43-item self-report questionnaire that measures schizotypal traits in the general population and contains four subscales relating to introvertive anhedonia, cognitive disorganization, unusual experiences and impulsive non-conformity. Responses (yes/no) are scored as 1 or 0 (eight reverse-scored items), with higher scores indicating greater schizotypal traits. The Finnish language translations showed comparably high internal consistency (Cronbach’s alpha coefficient: AQ = 0.711; O-LIFE = 0.842) to previous English language versions (Baron-Cohen *et al.*, 2001; Mason *et al.*, 2005).

### Biological motion stimulus

In the fMRI scanner, participants watched an audiovisual montage of 96 clips taken from popular movies with English speech (mean clip duration of 11.5 s and total duration of 19.6 min); the participants had no specific task other than to pay attention to the movie. The montage was designed to provide a high-dimensional representation of complex naturalistic social and emotional interactions and has been validated in previous studies (Lahnakoski *et al.*, 2012; Karjalainen *et al.*, 2017, 2019). The clips were presented in a fixed order for each participant to allow for the intersubject synchronization analysis. A fixation cross was presented at the start (5.2 s) and end (15.6 s) of the run. The stimulus video was displayed using goggles affixed to the head coil (NordicNeuroLab VisualSystem). The audio was played through SensiMetrics S14 earphones (100 Hz to 8 kHz bandwidth and 110 dB sound pressure level). The volume was adjusted individually to a comfortable level that could still be heard over the scanner noise.

**Fig. 1.**
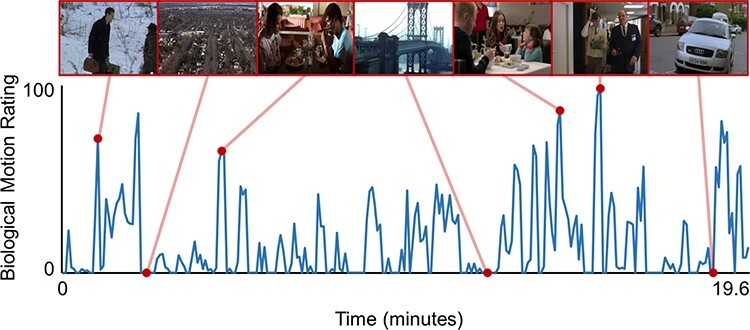
Average viewer ratings of the extent of biological motion in the stimulus. Representative frames are depicted at the top (in sequential order), with corresponding data points marked.

The montage was viewed by five separate neurotypical participants who provided a continuous rating for the presence of biological motion, reflecting the extent and frequency of movement (Figure 1). The stimulus was presented on a computer monitor and headphones, while the participant provided ratings by moving the mouse forward for the increased presence or backward for the decreased presence. Ratings were taken at 0.25 Hz intervals and downsampled to match the repetition time (TR) of the fMRI time series to be used as a regressor of interest to establish the relationship between biological motion perception and the magnitude and synchronization of neural activity. Interclass correlation analysis (*r *= 0.57) indicated moderate reliability between raters.

## Procedure

### MRI data acquisition and preprocessing

MRI scanning took place at Turku PET Centre, University of Turku, using a Phillips Ingenuity TF PET/MR 3T whole-body scanner. High-resolution (1 mm^3^) structural images were obtained with a T1-weighted sequence (TR 9.8 ms, TE 4.6 ms, flip angle 7^o^, 250 mm FOV and 256 × 256 reconstruction matrix). A total of 467 functional volumes were acquired, with a T2*-weighted echo-planar imaging sequence (TR 2600 ms, TE 30 ms, 75^o^ flip angle, 240 mm FOV, 80 × 80 reconstruction matrix, 62.5 kHz bandwidth, 3.0 mm slice thickness and 45 interleaved slices acquired in an ascending order without gaps).

Preprocessing of MRI data used fMRIPprep 1.3.0.2 (Esteban *et al.*, 2019). The anatomical T1-weighted reference image was subject to correction for intensity non-uniformity, skull stripping, brain surface reconstruction and spatial normalization to the ICBM 152 Nonlinear Asymmetrical template version 2009c (Fonov *et al.*, 2009) using non-linear registration with antsRegistration (ANTs 2.2.0) and brain tissue segmentation. The functional data were subject to coregistration to the T1w reference, slice-time correction, spatial smoothing with a 6-mm Gaussian kernel, automatic removal of motion artifacts using ICA-AROMA (Pruim *et al.*, 2015) and resampling of the MNI152NLin2009cAsym standard space. Low-frequency drifts were removed with a 240-s Savitzky–Golay filter (Çukur *et al.*, 2013).

### Data analysis

All participants were scanned sequentially, and data analysis proceeded on the individualized time series. We conducted both GLM and intersubject phase synchronization (ISPS) analyses to establish how biological motion perception is associated with regionally specific changes in BOLD activity and neural synchronization between individuals, respectively. In addition, we repeated these analyses with questionnaire scores as participant-level regressors to establish how BOLD activity and neural synchronization between individuals associated with biological motion perception vary with autistic and schizotypal traits.

Based on our hypotheses, we focused on two conjunctions of trait measures. First, the total AQ and O-LIFE scores were entered as orthogonalized regressors in the GLM and ISPS analyses. Second, the social skills and introvertive subscales of the AQ and O-LIFE, respectively, were entered as orthogonalized regressors. That is, in each analysis, the two scores were entered as regressors, with each acting as a regressor of no interest to the other. This enabled the investigation of the independent contributions of these traits to explain the neural response to biological motion despite the high covariance between the scores themselves. Further exploratory analyses using subscale scores within each trait measure (e.g. all subscales of the AQ and all of the O-LIFE) and inter-trait relationships between subscales revealed to be highly correlated [e.g. between Communication (AQ) and Impulsive Non-Conformity (O-LIFE)] can be found in the Supplementary Materials.

### GLM analysis

GLM analyses were conducted with SPM12 (www.fil.ion.ucl.ac.uk/spm) with a two-stage random-effects analysis. The biological motion ratings were convolved with a canonical hemodynamic response function and entered as a regressor into the first-level GLM analysis, using a high-pass filter of 128 s. The results of each participant in the first-level analysis were entered into a second-level random-effects analysis using a one-sample *t*-test, with a family-wise error (FWE) alpha threshold of *P *< 0.001. The questionnaire scores were entered as a participant-level regressor in the second-level analysis, with a cluster-level false discovery rate threshold after an uncorrected voxel threshold of *P *< 0.001.

### ISPS analysis

The data were preprocessed for phase synchronization analysis using the FunPsy toolbox (https://github.com/eglerean/funpsy, see Glerean *et al.*, 2012). For each participant, the voxel-specific time series was band-pass filtered (0.04–0.07 Hz), and the phase analytic signal (in radians) of the Hilbert transformed BOLD response of each voxel was calculated. The phase analytic signal at each timepoint was subtracted (and inversed) from that of the equivalent voxel from each of the other participants, and then averaged, to produce a 4D (space × time) measure of phase similarity of each participant with the rest of the sample. As the phase similarity measure is instantaneous, it provides a more temporally precise indicator of neural synchronization than the sliding window analyses of intersubject correlation.

Two ISPS analyses were conducted. (I) The relationship between ISPS and biological motion perception was investigated by taking each participant’s voxel-specific phase similarity time series and correlating it with the HRF-convolved biological motion regressor for each voxel. The *r* values were Fisher *z*-transformed and entered into a second-level analysis in SPM12 using a one-sample *t*-test and an FEW-corrected alpha threshold of *P *< 0.001. (II) The questionnaire scores were entered as a participant-level regressor to establish how neural synchronization (ISPS) associated with biological motion perception varies with autistic and schizotypal traits, with a cluster-level threshold after an uncorrected voxel threshold of *P *< 0.001.

## Results

### Questionnaire data

**Fig. 2.**
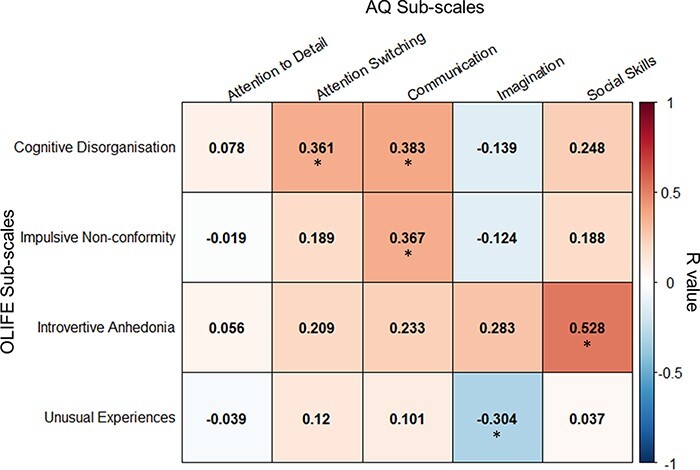
The inter-trait correlations between the subscales of the Autistic Spectrum Quotient and the Oxford–Liverpool Inventory of Feelings and Experiences. Correlations significant at Bonferroni-corrected *P *< 0.0025 are marked with an asterisk.

Descriptive statistics for the AQ and O-LIFE can be seen in Table 1. AQ scores were higher in males (*m *= 18.4, s.d. = 5.7) than females (*m *= 15.2, s.d. = 5.4, *t*(95) = 2.86, *P *= 0.005). There were no sex differences in O-LIFE scores (*t*(95) = 0.419, *P *= 0.676). Age did not correlate with scores on either the AQ (*r *= −0.020, *P *= 0.843) or O-LIFE (*r *= −0.041, *P *= 0.690). The total autistic traits and total schizotypal traits were positively correlated [*r *= 0.305, *P *= 0.002, 95% confidence interval (CI) = 0.111–0.499, BF10 = 12.03] and so too were the social skills and introversion subscales that may contribute to phenotypic convergence (*r *= 0.528, *P *= 2.78e-08, 95% CI = 0.355–0.701, BF10 = 503 158.12). Figure 2 shows the full inter-trait subscale correlation matrix (Bonferroni *P *< 0.0025, see Supplementary Figure S1 for the full correlation matrix between all subscales).

**Table 1.** AQ and O-LIFE scores in total and for each subscale

		Mean (s.d.)
AQ	Total	16.8 (5.8)
	Attention to detail	4.6 (2.1)
	Attention switching	4.2 (2)
	Communication	2.2 (1.8)
	Imagination	2.8 (1.8)
	Social skills	3.1 (2)
O-LIFE	Total	11.2 (6.6)
	Cognitive disorganization	4.1 (2.9)
	Impulsive non-conformity	2.2 (2)
	Introvertive anhedonia	2.2 (1.8)
	Unusual experiences	2.8 (2.6)

### The relationship between neural activity and biological motion perception

**Fig. 3.**
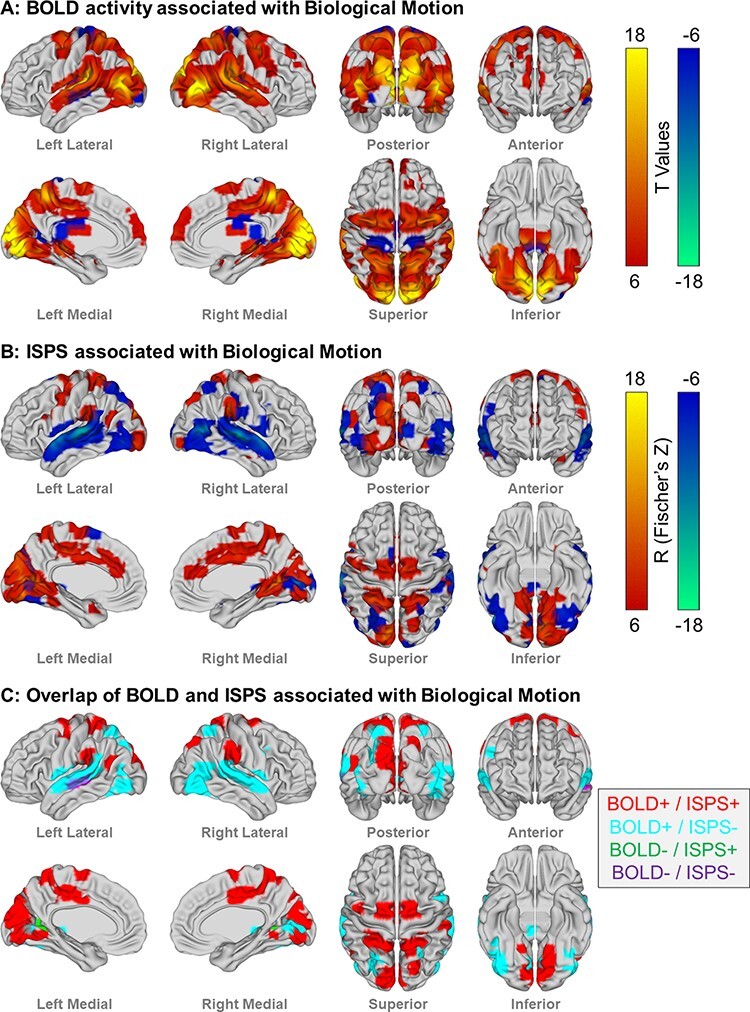
The convergent and divergent patterns of neural activity and synchronization associated with biological motion. (A) Regions exhibiting an increase (red) or decrease (blue) in neural activity using GLM analyses with biological motion as a regressor (FWE *P *< 0.001). (B) Regions exhibiting an increase (red) or decrease (blue) in intersubject neural synchronization associated with biological motion (FWE *P *< 0.001). (C) Logical overlays of regions exhibiting both neural activity and synchronization associated with biological motion, with relationships being convergent (positive or negative for both neural activity and synchronization) or divergent (positive and negative for either neural activity or synchronization).

The biological motion was associated with a widespread and distributed increase in neural activity (Figure 3A, FWE *P *< 0.001, Supplementary Tables S1 and S2). The bilateral activation was evident in the lingual gyri; cuneus; precuneus; thalamus; precentral gyrus; superior, medial and inferior frontal gyri; superior, middle and inferior temporal gyri; fusiform gyri; and lentiform nucleus. Unilateral activation was evident in the right superior and inferior frontal gyri. Cerebellar activation was observed in the bilateral cerebellar tonsil, uvula of vermis and right declive. Negative relationships between biological motion and neural activity were found in the bilateral caudate/parahippocampus, bilateral post-central gyri, right thalamus and bilateral middle insula.

### The relationship between neural synchronization and biological motion perception

The biological motion was associated with an increase in ISPS in a similarly distributed but more discrete set of regions (Figure 3B, FWE *P *< 0.001, Supplementary Tables S3 and S4) than was observed in the GLM analysis. Bilateral increases were observed in the lingual gyri, inferior parietal lobes, superior parietal lobes, middle temporal gyri and anterior cingulate gyri. Unilateral synchronization in the left hemisphere was observed in the cuneus, fusiform gyrus, precentral gyrus, superior frontal gyrus and inferior frontal gyrus and in the right hemisphere in the inferior occipital gyrus, precuneus, post-central gyrus, parahippocampus, posterior cingulate, middle cingulate gyrus, medial-frontal gyrus, claustrum, vulva of vermis and right declive. ISPS was negatively associated with biological motion bilaterally in the lingual gyri, fusiform gyri, cuneus, superior and middle temporal gyri and superior parietal lobes. Right hemispheric decreases were evident in the pre- and post-central gyri, inferior parietal lobe, thalamus, parahippocampus and inferior frontal gyrus. Left hemispheric decreases were evident in the inferior and middle occipital gyri, inferior temporal gyrus, precuneus, posterior cingulate and superior frontal gyrus.

### Overlapping neural activity and synchronization associated with biological motion perception

Logical overlays of the maps generated by the GLM and ISPS analyses reveal the convergent and divergent patterns of neural activity and synchronization associated with biological motion perception (Figure 3C).

#### GLM positive/ISPS positive

Both an increase in neural activity and ISPS were evident bilaterally in the precuneus, superior parietal lobe, precentral gyrus, cuneus/lingual gyri, posterior cingulate gyrus, inferior parietal lobe and left middle temporal gyrus and right claustrum.

#### GLM positive/ISPS negative

Several regions exhibited an increase in neural activity but a decrease in ISPS in response to biological motion, most notably in a large bilateral swathe along the superior temporal gyri, and also bilateral superior parietal lobe, bilateral fusiform gyrus, left cuneus and precuneus, right lingual gyrus, right middle temporal gyrus and right inferior frontal gyrus.

#### GLM negative/ISPS positive

Two small regions in the bilateral parahippocampus exhibited a decreased neural activity and an increased ISPS associated with biological motion perception.

#### GLM negative/ISPS negative

A prominent region in the left middle temporal gyrus exhibited both a decreased neural activity and ISPS with biological motion perception, as did small regions in the left posterior cingulate and right parahippocampus.

### Autistic and schizotypal traits associated with neural activity in response to biological motion

**Fig. 4.**
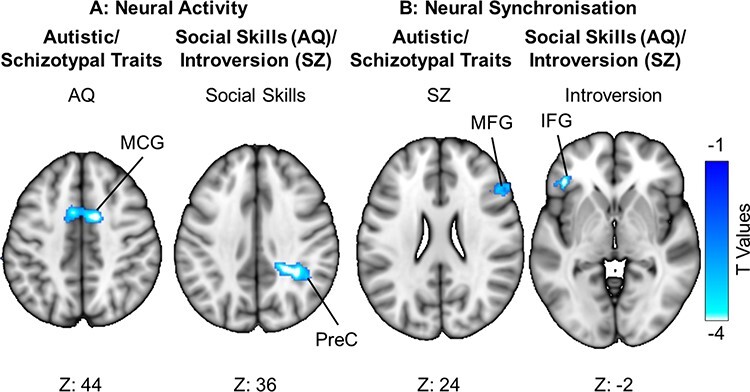
Patterns of neural activity and synchronization associated with biological motion, which dissociate autistic and schizotypal traits. (A) The neural activity associated with biological motion decreased with increasing autistic traits (with schizotypal traits as an orthogonal regressor) in the middle cingulate gyrus (MCG) and decreased with increasing atypical social skills (with introversion as an orthogonal regressor) in the precuneus (PreC). (B) Neural synchronization associated with biological motion decreased with increasing schizotypal traits (with autistic traits as an orthogonal regressor) in the middle frontal gyrus (MFG) and decreased with increasing introversion (with social skills as an orthogonal regressor) in the inferior frontal gyrus (IFG). For visualization purposes, these figures depict an uncorrected voxel threshold of *P *< 0.01, followed by an FWE cluster threshold of *P *< 0.05.

We next conducted a GLM analysis to establish the extent to which autistic and schizotypal traits are associated with neural activity in response to biological motion (Figure 4A). The first-level analyses with biological motion as a regressor were entered into a second-level analysis with trait scores as a regressor. Autistic traits, with schizotypal traits as a covariate, were negatively correlated with the neural response to biological motion in a cluster (*k* = 187) with two peak voxels in the left and right middle cingulate gyrus. The AQ subscale of Social Skills, with the O-LIFE subscale of Introversion entered as a covariate, was negatively associated with the neural response to biological motion in a cluster (*k* = 157) with two peak voxels in the right precuneus. The converse analyses showed that neural activity associated with biological motion perception was associated neither with schizotypal traits (with autistic traits as a covariate) nor with the Introversion subscale (with the Social Skills subscale as a covariate).

### Autistic and schizotypal traits associated with neural synchronization in response to biological motion

The relationship between ISPS and biological motion decreased with schizotypal traits, with autistic traits as a covariate, in a cluster (*k* = 39) with two peak voxels in the right middle frontal gyrus. The O-LIFE subscale of Introversion, with the AQ subscale of Social Skills as a covariate, was negatively associated with the relationship between ISPS and biological motion in a cluster (*k* = 39) with a peak voxel in the left inferior frontal gyrus (Figure 4B). The converse analyses showed that neural synchronization in response to biological motion perception was associated neither with autistic traits (with schizotypal traits as a covariate) nor with the Social Skills subscale (with the Introversion subscale as a covariate).

## Discussion

While neural activity associated with the perception of biological motion increases in a widespread network of brain regions, the synchronization of neural activity between individuals differs within this network. Biological motion perception was associated with increased neural synchronization in the visual and parietal regions, whereas desynchronization was evident in the temporal and frontal regions. Autistic traits are associated with changes in the overall neural activity related to biological motion in the precuneus and middle cingulate gyrus, while schizotypal traits are associated with changes in neural synchronization in the middle and inferior frontal gyri. These results suggest that correlated autistic and schizotypal traits, especially those relating to social behavior, are associated with different neural responses to social stimuli, namely the magnitude of neural activity for autistic traits, and the between-individual synchronization of neural activity for schizotypal traits.

### Convergent and divergent patterns of neural activity and synchronization in relation to biological motion perception

The perception of biological motion was associated with an increase in neural activity in an extensive network of regions encompassing the occipital, temporal, parietal and frontal cortices, characterizing the well-established ‘social brain’ implicated in action observation (occipital cortex, posterior temporal regions and parietal and pre-motor regions) and mentalizing (anterior-temporal, temporal-parietal junction and medial-frontal gyri) (Adolphs, 2009; Li *et al.*, 2018). However, the distribution of neural synchronization between individuals during biological motion perception varied within this network. Regions in primary visual areas, face- and body-selective visual areas, inferior parietal lobe, precentral gyrus, temporal-parietal junction and cingulate cortex showed an increase in neural activity that is also highly synchronized across individuals. In contrast, the superior parietal lobe, superior and middle temporal gyri, fusiform gyri and inferior frontal gyri exhibited desynchronization of neural activity despite an overall increase in neural activity. Moreover, several regions exhibited either increases or decreases in synchronization despite no change in the overall neural activity. These regions may be involved in the perception of stimulus features that are not necessarily social in nature, such as motion itself, or subjective interpretations of the intentions and context that are informed by biological motion. These results highlight that analysis of intersubject similarity of the neural response provides complementary information by identifying regions that would not otherwise have been associated with the perception of social stimuli.

Broadly speaking, neural synchronization varied along a posterior–anterior axis, with increased synchronization observed in posterior visual areas and the parietal lobe, whereas decreased synchronization was observed in temporal association regions and frontal regions. This agrees with previous findings of a systematic gradient of neural reliability (Kauppi *et al.*, 2010, 2017) that reflects a global cortical hierarchy of parsing, integration and prediction of information at different timescales (Hasson *et al.*, 2008, 2010, 2015; Kiebel *et al.*, 2008; Baldassano *et al.*, 2017; Huntenburg *et al.*, 2018). Disturbances in the extent of this gradient have also been observed in autism (Watanabe *et al.*, 2019) and psychosis (Wengler *et al.*, 2020). This dichotomy may provide a key insight into the neural mechanisms underpinning biological motion perception. Sensorimotor areas operate at short timescales and are tightly coupled to stimulus features (e.g. moment-to-moment changes in visible body parts, the extent of motion or the specific action), therefore showing a high degree of neural reliability across subjects. Neural activity is less reliable in temporal and prefrontal regions (despite an overall increase in activity in the BOLD–GLM analysis), which operate at longer timescales and are involved in idiosyncratic and subjective interpretations and predictions of other’s behaviors and decisions about how to act in response to this.

### Neural activity and synchronization differentiate individual differences in autistic and schizotypal traits

Autistic and schizotypal traits were positively correlated, especially on measures of social behavior, and also on more general indices of cognitive organization and control. Only traits relating to imagination and unusual experiences (homologous to positive schizotypal traits) were negatively correlated. These findings support previous theoretical and empirical work suggesting an intersection of features of autism and schizophrenia and related traits in the neurotypical population (Russell-Smith *et al.*, 2011; Zhou *et al.*, 2019; Isvoranu *et al.*, 2021). The divergent pattern of autism- and schizotypy-dependent neural activity and synchronization in response to biological motion perception suggests that these traits reflect a phenotypic overlap with separate neural bases.

Autistic traits, and social skills in particular, were associated with a decrease in the overall neural activity related to biological motion, whereas schizotypal traits, and introversion in particular, were associated with a decrease in neural synchronization. The social difficulties associated with autism are, in part, reflected in reduced overall neural activity in response to the perception of complex social-emotional interactions, which may reflect or cause an insensitivity to such stimuli compared to those with schizophrenia (Sasson *et al.*, 2007). However, while watching those same interactions, the social difficulties associated with schizophrenia correspond to decreased neural synchronization between individuals, which may reflect not only a reduction in short-term integration of stimulus features but also an inability to establish neural ‘rapport’ with others that enables mutual psychological states and may also contribute to spurious mental state attributions (Ciaramidaro *et al.*, 2015). Moreover, the decreased synchronization associated with schizotypal traits was focused in frontal areas (the middle and inferior frontal gyri), which are associated in schizophrenia with high-level inferences and decision-making in social interactions (Russell *et al.*, 2000; Li *et al.*, 2012; Takei *et al.*, 2013; Shin *et al.*, 2015). In contrast, decreased neural activity associated with autistic traits was observed in middle cingulate gyrus and precuneus, both of which have been implicated in autism with a reduced awareness of one’s own actions and decisions in social interactions and distinguishing self from others (Tomlinm *et al.*, 2006; Chiu *et al.*, 2008; Lombardo *et al.*, 2010; Martineau *et al.*, 2010; Just *et al.*, 2014). These regions were not associated with the perception of biological motion at the population level (see also Puglia and Morris, 2017), suggesting that social difficulties experienced by autistic people and those diagnosed with schizophrenia may result from downstream or upstream secondary processes that rely on, but are not directly implicated in, the perception of other’s behavior.

## Limitations

Autistic and schizotypal traits in the neurotypical population can be assessed in isolation, free of differences in cognitive development and neurodevelopmental or mental health conditions that are associated with those who have received a diagnosis of autism or schizophrenia. Furthermore, it is possible to establish how neural activity correlates with variation in these traits in a large sample, which is not possible when looking at qualitative differences between diagnosed groups (Landry and Chouinard, 2016). Nevertheless, the spectrum models of autism and psychosis assume that these traits are conceptually aligned with these conditions in a linear and unidimensional distribution (Sasson and Bottema-Beutel, 2021). Autism and psychosis-spectrum disorders are multidimensional and exhibit qualitative differences in the traits measured in these surveys. The extent to which the current results can be extrapolated either quantitatively or qualitatively to those with autism or schizophrenia remains to be seen.

Furthermore, although we aimed to reflect naturalistic conditions by having participants freely and passively view a complex and dynamic social stimulus, it is possible to interpret neither the functional significance of neural (de)synchronization associated with biological motion perception nor the activity of regions in which the neural profile was associated with schizotypal and autistic traits. These regions have been previously implicated in socio-cognitive performance in autism and schizophrenia, but how their differing neural profiles contribute to convergent behavioral phenotypes requires controlled experimental manipulation. Moreover, as eye movements were not measured, it is not possible to assess the differing attention resources allocated to the stimulus and how these may be associated with autistic and schizotypal traits (although attentional and cognitive subscales of these trait measures did not correlate with those relating to social behavior and were associated with different neural responses, as reported in the Supplementary Material).

Lastly, the neurophenotypic profile of those providing the ratings of biological motion was not recorded. Future studies should ensure that the ratings reflect a similar variation in the neurotypical population as the neurological data to which they are compared.

## Conclusion

The perception of biological motion elicits both overlapping and dissociable patterns of neural activity and neural synchronization between individuals. Increases in neural synchronization were observed primarily in regions associated with stimulus processing (visual and motor regions), whereas decreases in neural synchronization were observed primarily in regions associated with interpretation and decision-making (temporal and frontal regions). These differences correspond to a well-established posterior–anterior axis of neural reliability implicated in temporal parsing, integration and prediction, which we can now apply to the perception of social interactions. Moreover, patterns of neural activity and synchronization differentiated the highly correlated individual differences in autistic and schizotypal traits in a large sample of the neurotypical population in regions that were not directly implicated in biological perception but which have previously been implicated in social functions. The highly convergent individual differences in social behavior that correspond to autistic and schizotypal traits, and by possible extension the common social difficulties encountered by those with autism and schizophrenia themselves, do not reflect a shared etiology but disparate mechanisms that elicit superficially similar phenotypes. The use of complex and naturalistic social interactions provides new avenues for future research. Different temporal profiles of neural activity can be dissociated by the perception of other people’s behavior, and this can reveal different neural mechanisms associated with autistic and schizotypal individual differences that cannot be distinguished at the behavioral level. Interpersonal synchronization, at the behavioral or neural level, may provide the basis for the reciprocal and coordinated interactions upon which social functioning ultimately relies and that are inordinately affected in different ways across numerous diagnoses (Schilbach, 2016). Establishing heterogeneity within conditions and homogeneity between conditions in the extent to which social functioning is affected, and the neurocognitive mechanisms that underpin them, may serve to identify difficulties and interventions more specifically than traditional nosological descriptions (Cuthbert and Insel, 2013; Morris *et al.*, 2022).

## Supplementary Material

nsad011_Supp

## Data Availability

Thresholded and unthresholded results of the GLM analyses and inter-subject phase synchronization analyses are available on NeuroVault (https://identifiers.org/neurovault.collection:12349). Questionnaire data for the AQ and OLIFE (totals and subscales) are available at https://doi.org/10.17605/OSF.IO/VG8ZX. All code is available from the authors upon request.
