# Supplementary material for: Neural responses to biological motion distinguish autistic and schizotypal traits
Source: Soc Cogn Affect Neurosci. 2023 Feb 27;18(1):nsad011. doi: 10.1093/scan/nsad011 (PMC10032360; doi:10.1093/scan/nsad011)
Supplement: nsad011_Supp [file nsad011_supp.zip › scan-22-197-File006.docx]

**Supplementary Materials**


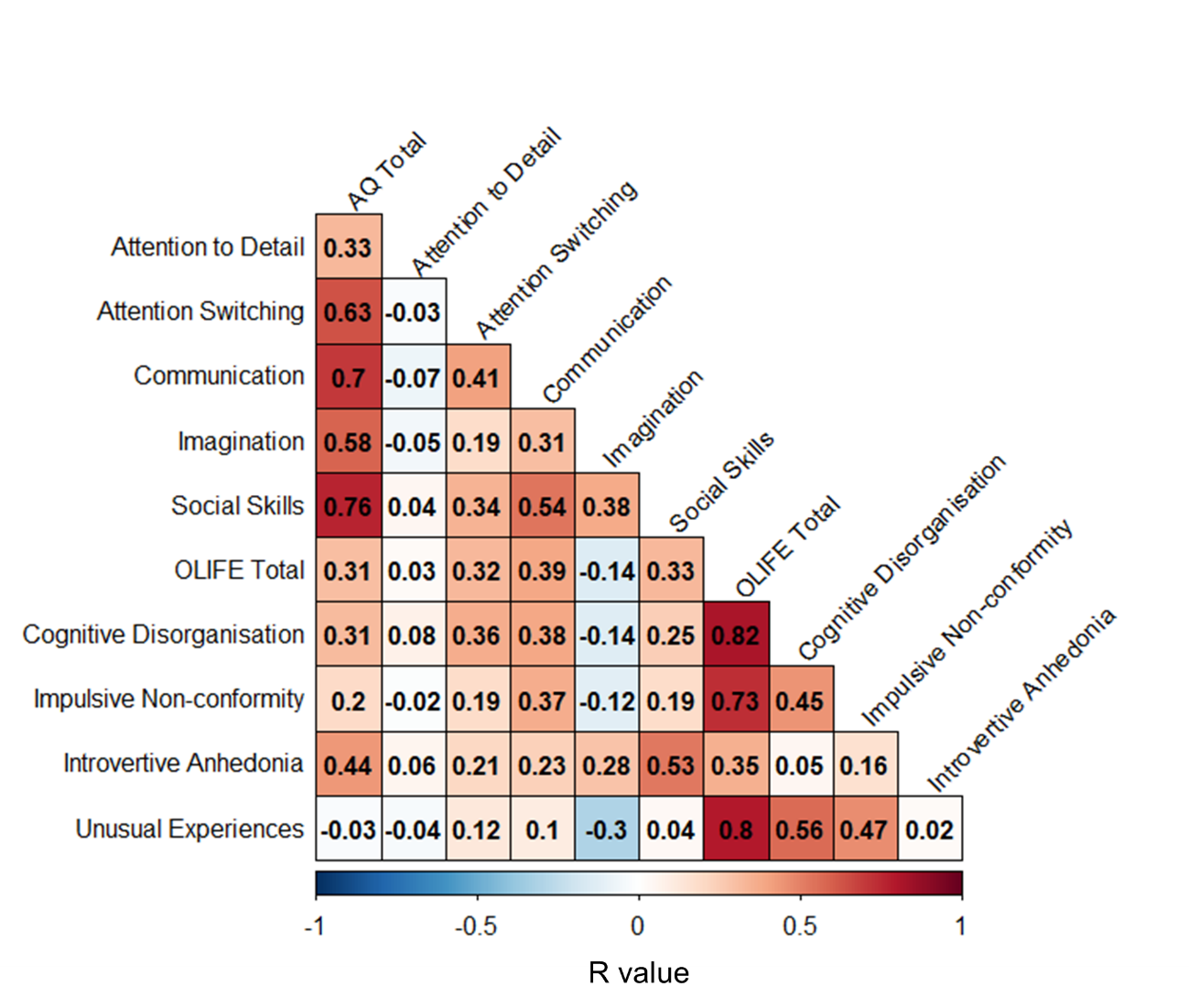


**Supplementary Figure 1.** Correlation matrix showing the within trait and between trait relationships between overall autistic and schizotypal traits and the respective sub-scales.

**Supplementary Tables**

**The relationship between neural activity and biological motion perception**

**Supplementary Table 1.** Neural activity positively associated with Biological Motion Perception (Figure 3A). Cluster sizes and Peak T values are reported in MNI coordinate space. Voxel level FWE alpha threshold of *p*<.001.

| Cluster Size | Peak T value | X | Y | Z | MNI Region |
| --- | --- | --- | --- | --- | --- |
| 20758 | 24.26 | 10 | -82 | 2 | r lingual gyrus |
|  | 23.64 | 8 | -78 | -6 | r lingual gyrus |
|  | 23.44 | 6 | -90 | 8 | r lingual gyrus |
| 6600 | 19.13 | 8 | -50 | 58 | r precuneus |
|  | 16.68 | -6 | -50 | 60 | r precuneus |
|  | 14.99 | -14 | -24 | 40 | l cingulate gyrus |
| 1169 | 15.5 | -14 | -30 | -4 | l thalamus |
|  | 14.21 | 12 | -30 | -2 | l thalamus |
|  | 14.13 | -6 | -34 | -6 | r culmen |
| 152 | 14.25 | 16 | -52 | -52 | r cerebellar tonsil |
| 1978 | 14.22 | 46 | 0 | 54 | r precentral gyurs |
|  | 12.36 | 30 | -4 | 50 | r precentral gyurs |
|  | 11.33 | 32 | -4 | 66 | r precentral gyurs |
| 383 | 13.29 | -14 | -56 | -52 | l cerebellar tonsil |
|  | 10.18 | 0 | -60 | -38 | l cerebellar tonsil |
|  | 6.74 | -12 | -58 | -40 | l cerebellar tonsil |
| 1349 | 12.63 | -40 | -6 | 54 | l precentral gyrus |
|  | 11.78 | -30 | -8 | 54 | l precentral gyrus |
|  | 11.41 | -22 | -6 | 70 | l precentral gyrus |
| 16 | 10.13 | 4 | -74 | -34 | unvula of vermis |
|  | 6.66 | -4 | -72 | -34 | unvula of vermis |
| 284 | 9.22 | 6 | 64 | 14 | r medial frontal gyrus |
|  | 7.37 | 2 | 56 | 32 | r medial frontal gyrus |
|  | 6.43 | 4 | 62 | 24 | r superior frontal gyrus |
| 140 | 9.16 | -46 | -56 | -20 | l fusiform gyrus |
|  | 8.21 | -44 | -68 | -18 | l fusiform gyrus |
| 59 | 8.17 | 36 | -50 | -36 | r cerebellar tonsil |
| 32 | 8 | 36 | -66 | -22 | r declive |
|  | 6.53 | 40 | -56 | -26 | r declive |
| 25 | 7.37 | 24 | 42 | 50 | r superior frontal gyrus |
| 26 | 7.36 | 22 | 14 | 10 | r lentiform nucleus |
|  | 7.14 | 24 | 12 | 2 | r lentiform nucleus |
| 37 | 7.29 | 40 | 14 | 24 | r inferior frontal gyrus |
| 11 | 6.79 | -24 | 16 | 2 | l lentiform nucleus |

**Supplementary Table 2.** Neural activity negatively associated with Biological Motion Perception (Figure 3A). Cluster sizes and Peak T values are reported in MNI coordinate space. Voxel level FWE alpha threshold of *p*<.001.

| Cluster Size | Peak T value | X | Y | Z | MNI Region |
| --- | --- | --- | --- | --- | --- |
| 411 | 13.16 | 22 | -42 | 20 | r caudate nucleus |
|  | 12.72 | 28 | -48 | 14 | r caudate nucleus |
|  | 12.18 | 34 | -52 | 4 | r parrahippocampus |
| 321 | 12.3 | -30 | -58 | 6 | l parrahippocampus |
|  | 11.37 | -20 | -46 | 16 | l caudate nucleus |
|  | 11.09 | -10 | -22 | 26 | l caudate nucleus |
| 165 | 9.11 | -24 | -30 | 68 | l post central gyrus |
|  | 8.62 | -16 | -32 | 70 | l post central gyrus |
| 40 | 8.46 | 2 | -32 | 16 | corpus collosum |
|  | 8.06 | 10 | -34 | 10 | r thalamus |
|  | 6.95 | 16 | -38 | 6 | r parrahippocampus |
| 114 | 8.45 | 18 | -32 | 68 | r post central |
|  | 8.01 | 24 | -30 | 62 | r post central |
| 18 | 7.93 | -38 | -8 | 18 | l middle insula |
| 12 | 7.68 | 8 | -4 | 26 | corpus collosum |
| 25 | 7.65 | 44 | -14 | 20 | r middle insula |
| 61 | 7.53 | -54 | -18 | -6 | l middle temporal gyrus |
|  | 6.56 | -54 | -26 | -2 | l middle temporal gyrus |
|  | 6.37 | -60 | -32 | 2 | l middle temporal gyrus |
| 11 | 6.7 | -40 | -16 | 20 | l insula |

**The relationship between neural synchronization and biological motion perception**

**Supplementary Table 3.** Neural synchronisation positively associated with Biological Motion Perception (Figure 3B). Cluster sizes and Peak T values are reported in MNI coordinate space. Voxel level FWE alpha threshold of p<.001.

| Cluster Size | Peak T value | X | Y | Z | MNI Region |
| --- | --- | --- | --- | --- | --- |
| 3147 | 15.68 | -2 | -80 | 10 | l cuneus |
|  | 13.83 | -12 | -80 | -6 | l lingual gyrus |
|  | 13.31 | -14 | -92 | 30 | l cuneus |
| 670 | 12.73 | 28 | -60 | 8 | r lingual |
|  | 12.52 | 24 | -52 | 4 | r parrahippocampus |
|  | 12.22 | 18 | -62 | 10 | r pos cingulate |
| 262 | 12.31 | 56 | -30 | 34 | r inferior parietal lobe |
|  | 8.67 | 64 | -30 | 24 | r inferior parietal lobe |
|  | 7.18 | 68 | -34 | 44 | r inferior parietal lobe |
| 515 | 11.77 | -14 | -26 | 42 | l cingulate gyrus |
|  | 9.78 | -12 | -18 | 42 | l cingulate gyrus |
|  | 9.7 | 6 | -12 | 42 | r cingulate gyrus |
| 526 | 10.93 | -32 | -46 | 70 | l superior parietal lobule |
|  | 10.51 | -18 | -54 | 74 | l superior parietal lobule |
|  | 9.97 | -12 | -50 | 62 | l superior parietal lobule |
| 203 | 10.3 | 8 | -10 | 74 | r medial frontal gyrus |
| 174 | 9.98 | -58 | -34 | 28 | l inferior parietal lobe |
| 305 | 9.92 | 8 | -52 | 74 | r post central gyrus |
|  | 9.1 | 28 | -44 | 72 | r post central gyrus |
|  | 9.03 | 20 | -54 | 76 | r superior pariteal lobe |
| 36 | 9.75 | 22 | -76 | 44 | r precuneus |
| 52 | 8.79 | 0 | -64 | -32 | uvulvua of vermis |
| 204 | 8.59 | -14 | -10 | 68 | l precentral gyrus |
|  | 8.59 | -14 | -8 | 76 | l superior frontal gyurs |
|  | 7.49 | -8 | -14 | 78 | l superior frontal gyurs |
| 39 | 8.53 | -46 | -6 | 58 | l precentral gyrus |
| 51 | 8.43 | -56 | -66 | 24 | l middle temporal gyrus |
|  | 6.59 | -50 | -62 | 20 | l middle temporal gyrus |
| 97 | 8.34 | 2 | 36 | 22 | r antior congulate |
|  | 7.63 | -10 | 36 | 24 | l anterior cingulate gyrus |
|  | 7.36 | 8 | 46 | 20 | r medial frontal gyrus |
| 34 | 8.22 | 14 | -38 | 48 | r precuneus |
| 13 | 8.22 | -8 | 22 | 36 | l anterior cingulate gyrus |
| 30 | 8.18 | 42 | -14 | 0 | r claustrum |
| 20 | 8.15 | -56 | 2 | 44 | l precentral gyrus |
|  | 6.93 | -48 | 4 | 44 | l precentral gyrus |
| 17 | 8.08 | -58 | 16 | 28 | l inferior frontal gyrus |
| 12 | 8.03 | -28 | 10 | -22 | l inferior frontal gyrus |
| 13 | 7.93 | 34 | -90 | -6 | inferior occipital gyrus |
| 44 | 7.84 | 6 | -80 | -8 | r declive |
|  | 7.37 | 8 | -86 | -14 | r declive |
| 16 | 7.75 | 4 | 8 | 34 | r middle cingulate gyrus |
| 16 | 7.73 | -30 | -40 | -14 | l fusiform gyrus |
| 16 | 7.5 | 16 | -76 | -22 | r declive |
| 10 | 7.35 | 16 | -46 | 58 | r precuneus |
| 28 | 7.3 | 6 | 22 | 36 | r middle cingulate gyrus |
|  | 7.01 | 0 | 16 | 40 | r middle cingulate gyrus |
| 10 | 6.97 | 52 | -8 | -20 | r middle temporal gyrus |

**Supplementary Table 4.** Neural synchronisation negatively associated with Biological Motion Perception (Figure 3B). Cluster sizes and Peak T values are reported in MNI coordinate space. Voxel level FWE alpha threshold of p<.001.

| Cluster Size | Peak T value | X | Y | Z | MNI Region |
| --- | --- | --- | --- | --- | --- |
| 2594 | 16.86 | -56 | 0 | -4 | l superior temporal gyrus |
|  | 16.65 | -60 | -20 | 2 | l superior temporal gyrus |
|  | 16.06 | -64 | -28 | 8 | l superior temporal gyrus |
| 1838 | 15.99 | 64 | -22 | 0 | r superior temporal gyrus |
|  | 15.11 | 68 | -2 | -2 | r superior temporal gyrus |
|  | 15.05 | 50 | -30 | 8 | r superior temporal gyrus |
| 410 | 14.9 | 48 | -62 | 10 | r middle temporal gyrus |
| 138 | 14.05 | 18 | -82 | 6 | r lingual gyrus |
| 155 | 11.48 | -22 | -72 | 36 | l precuneus |
|  | 9.1 | -24 | -74 | 24 | l precuneus |
|  | 7.04 | -26 | -80 | 46 | l precuneus |
| 21 | 10.97 | 34 | -44 | 0 | r parrahippocampus |
| 54 | 10.32 | -24 | -64 | 56 | l superior parietal lobe |
|  | 8.54 | -34 | -60 | 62 | l superior parietal lobe |
| 176 | 10.26 | -42 | -76 | -6 | l inferior occipital gyrus |
|  | 8.38 | -48 | -82 | -10 | l middle occipital cortex |
|  | 7.81 | -46 | -90 | -2 | l inferior occipital gyrus |
| 22 | 9.29 | 46 | -44 | -10 | r fusiform gyrus |
| 11 | 8.69 | 4 | -34 | -2 | r thalamus |
| 52 | 8.56 | -34 | -64 | -12 | l fusiform gyrus |
|  | 7.82 | -40 | -58 | -12 | l fusiform gyrus |
| 12 | 8.5 | -18 | -48 | 16 | l posterior cingulate |
| 11 | 8.48 | 66 | -18 | 30 | r postcentral gyrus |
| 148 | 8.48 | 42 | -72 | -14 | r fusiform gyrus |
|  | 8.46 | 50 | -62 | -12 | r fusiform gyrus |
|  | 7.7 | 38 | -80 | -10 | r fusiform gyrus |
| 11 | 8.44 | 14 | -102 | 8 | r cuneus |
| 14 | 8.1 | -18 | -72 | 50 | l cuneus |
| 14 | 8.05 | 56 | 6 | 14 | r inferior frontal gyrus |
| 21 | 7.87 | 52 | -30 | 44 | r inferior parietal lobe |
| 16 | 7.74 | -4 | 6 | 68 | l superior frontal gyrus |
| 17 | 7.61 | -2 | -70 | 4 | l lingual gyrus |
| 10 | 7.6 | -70 | -50 | 2 | l middle temporal gyrus |
| 22 | 7.58 | -54 | -78 | 2 | l inferior temporal gyrus |
|  | 7.28 | -52 | -76 | 12 | l middle temporal gyrus |
| 12 | 7.12 | 32 | -62 | 56 | r superior parietal lobe |
| 11 | 7.02 | 52 | 8 | 34 | r precentral gyrus |

**Supplementary Table 5.** Autistic and Schizotypal traits associated with neural activity and synchronization in response to biological motion (Figure 4). Cluster sizes and Peak T values are reported in MNI coordinate space. Cluster level FDR threshold after an uncorrected voxel threshold of *p*<.001.

| Analysis | Regressor (covariate) | Cluster Size | Peak T value | X | Y | Z | MNI Region |
| --- | --- | --- | --- | --- | --- | --- | --- |
| Neural Activity | Autistic Traits | 187 | 4.63 | 12 | 2 | 44 | r middle cingulate gyrus |
| Fig 4A | (Schizotypal traits) |  | 4.05 | -6 | 6 | 44 | l middle cingulate gyrus |
|  | Social Skills | 157 | 5.23 | 34 | -46 | 34 | r precuneus |
|  | (Introversion) |  | 4.91 | 26 | -42 | 36 | r precuneus |
| Neural Synchronization | Schizotypal Traits | 39 | 4.46 | 58 | 26 | 28 | r middle frontal gyrus |
| Fig 4B | (Autistic Traits) |  | 3.75 | 52 | 26 | 22 | r middle frontal gyrus |
|  | Introversion | 39 | 4.63 | -42 | 30 | -2 | l inferior frontal gyrus |
|  | (Social Skills) |  |  |  |  |  |  |

**Supplementary Analysis**

The main hypotheses of this study predicted that there would be a positive corelation between autistic and schizotypal traits, especially with respect to those subscales measuring social behaviours, and that despite these positive relationships, the differential patterns of neural activity may dissociate these convergent traits. This is indeed what we report. We also found several other facets of autistic and schizotypal traits that showed inter-trait correlations. These were positive relationships between Cognitive Disorganisation, Attention Switching, and Communication, between Communication and Impulsive Non-Conformity, and a negative relationship between Imagination and Unusual Experiences. Although these did not measure social behaviours, we nevertheless conducted exploratory analyses to assess the degree to which these correlated traits may also be differentiated by patterns of neural activity and synchronisation.

We first report the extent of inter-subject phase synchronization of neural activity across the sample, independent of stimulus features. For this, the ISPS measure in each voxel was collapsed across the temporal dimension for each subject. We then report how this overall neural synchronization varies as a function of individual differences in autistic and schizotypal traits, following evidence of decreased neural synchronization between people in those diagnosed with either autism (Hasson et al., 2009; Salmi et al., 2009) or schizophrenia (Lerner et al., 2018; Mäntylä et al., 2018). The total AQ and OLIFE scores were entered as participant level regressors, as were the social skills and introvertive sub-scales. We then conducted exploratory analysis of the inter-trait and intra-trait relationships with neural synchronization, by entering all sub-scales for each trait in separate analyses, and then all inter-trait sub-scales that demonstrated a relationship in the analysis of the questionnaire data (as described above).

We then report exploratory analyses of whether the neural activity and synchronisation associated with biological motion perception could distinguish intra-trait relationships between the subscales, by entering all subscales for each trait measure in separate analyses, followed by the correlated inter-trait sub-scales of the AQ and OLIFE.

**Individual differences in autistic and schizoptyal traits in neural synchronisation**

Descriptive statistics for the extent of inter-subject phase synchronization of neural activity across the sample, independent of stimulus features, are depicted in Supplementary Figure 2. Neural synchronization was greatest in posterior visual areas and auditory regions in the temporal cortex, as would be expected given that all participants are viewing and listening to the same stimulus.

**
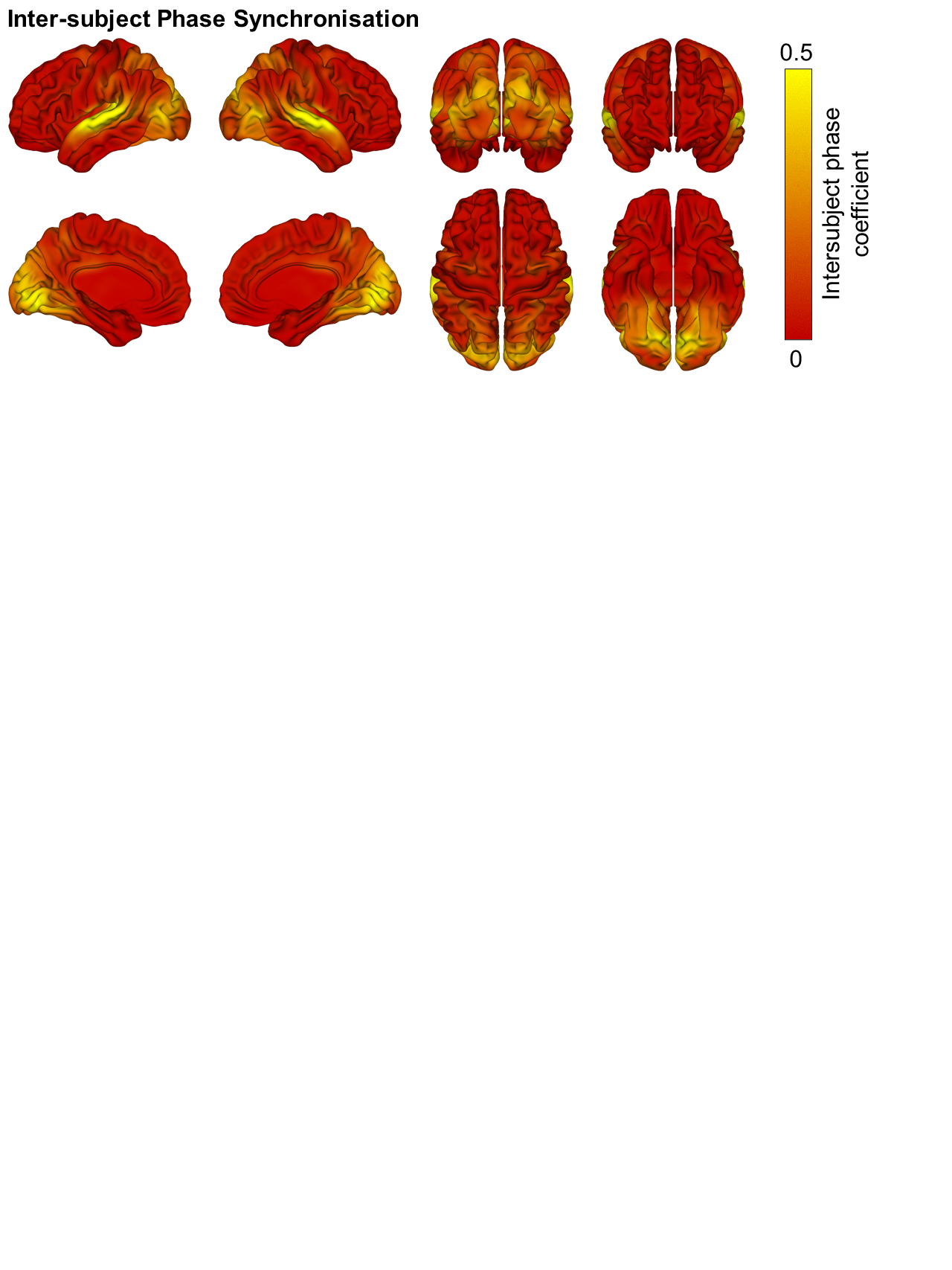
**

**Supplementary** **Figure 2.** Inter-subject phase synchronization of neural activity across the sample, independent of stimulus features.

Of interest is how this neural synchronization varies with autistic and schizotypal traits in general, and social skills and introversion specifically, across the whole stimulus run, irrespective of biological motion content. Autistic and schizotypal traits, which are positively correlated, exhibit divergent patterns of ISPS (Supplementary Figure 3), with autistic traits positively correlating with ISPS in a cluster (k = 86) with three peak voxels in the left superior temporal gyrus, and increasing schizotypal traits associated with a decrease in ISPS in a cluster (k = 64) with three peak voxels in the right precuneus. The increased synchronization with autistic traits in the superior temporal gyrus was observed by Salmi et al., (2009), and the decreased synchronization in the precuneus with increasing schizophrenic symptoms was observed by Lerner et al., (2018). It is unclear exactly how these differential patterns of neural synchronization contribute to autistic and schizotypal traits, however, the specificity of these replications warrants further investigation.

Despite a positive correlation between the Social Skills sub-scale of the AQ and the Introversion sub-scale of the OLIFE, neither were associated with ISPS when entered as regressors.

**
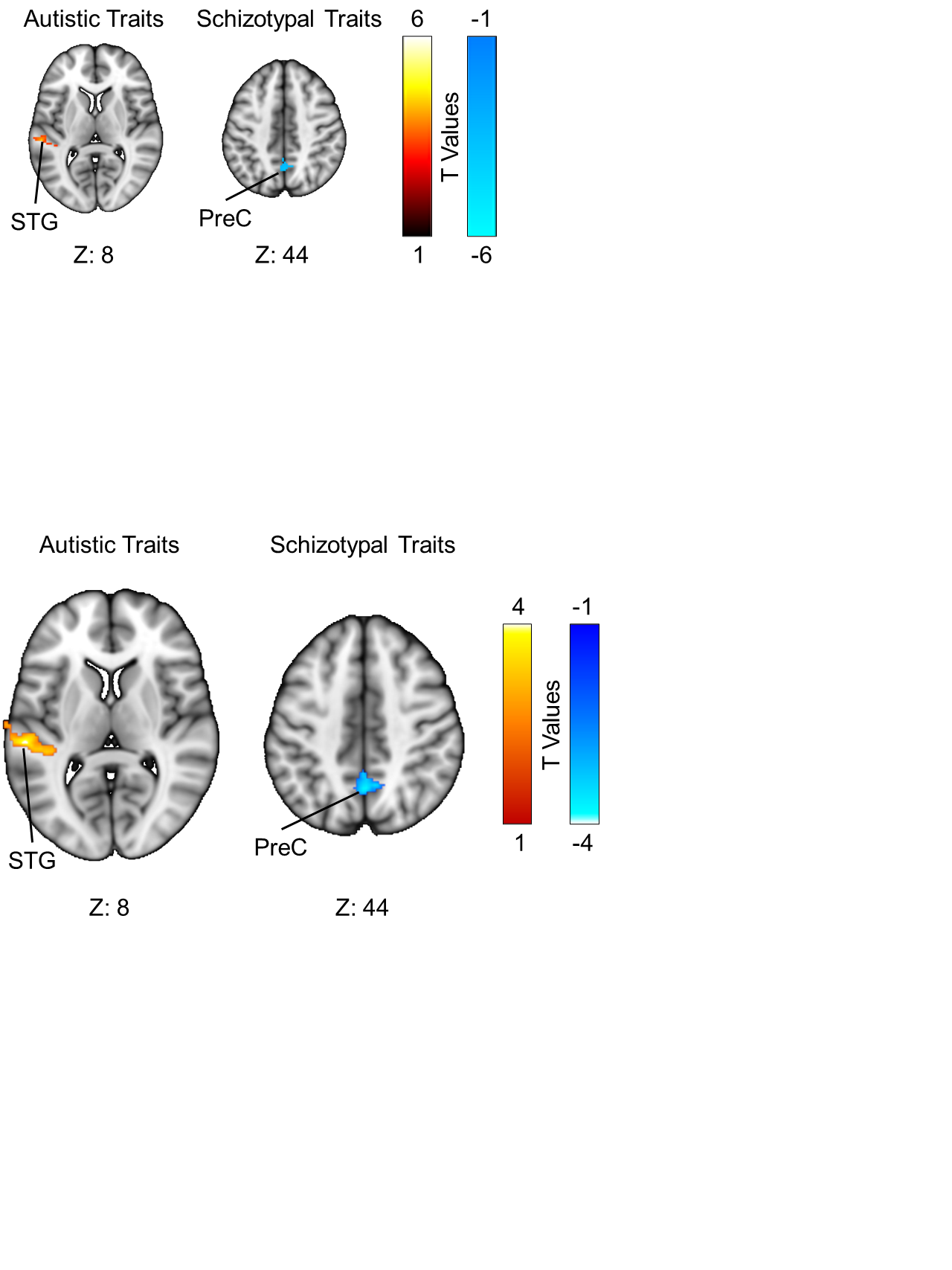
**

**Supplementary Figure 3.** The relationship between overall autistic and schizotypal traits and inter-subject phase synchronization independently of stimulus features. Autistic and schizotypal traits were entered as orthogonal regressors against the degree of inter-subject phase synchronization, across the stimulus run. Neural synchronization increased with increasing autistic traits in the Superior Temporal Gyrus (STG), and decreased with increasing schizotypal traits in the Precuneus (PreC). For visualization purposes, these figures depict an uncorrected voxel threshold of *p* < .01, followed by a FWE cluster threshold of *p* < .05.

*Intra-Trait analyses (p < 001, cluster corrected)*

*Autistic Traits (Supp Figure 4A):* The five sub-scales of the Autistic Spectrum Quotient were entered as regressors to establish if they were associated with the degree of IPS across the whole stimulus run, irrespective of biological motion content. Attention Switching was positively correlated with IPS in one cluster (k = 241) with peak voxels in the right posterior insula cortex and claustrum. IPS decreased with increasing Social Skills deficits in one cluster (k = 73) in the inferior semi-lunar lobe of the cerebellum.

*Schizotypal Traits (Supp Figure 4B):* The four sub-scales of the Oxford-Liverpool Inventory of Feelings and Experiences were entered as regressors to establish how individual differences in schizotypal traits are associated with IPS. Increasing traits of Impulsive Non-conformity were associated with decreasing IPS in one cluster (k = 100) with a peak voxel in the right precuneus. A decrease in IPS was also associated with increasing Unusual Experiences in two clusters (k = 72) with two peak voxels in the left cuneus, and three in the right precuneus.

*Inter-Trait analyses (p < 001, cluster corrected)*

The AQ sub-scale of Communication and the OLIFE sub-scale of Impulsive Non-conformity, whilst positively correlated, revealed different patterns of IPS (Supp Figure 4C). Communication difficulties were associated with increased IPS in two clusters (k = 70) with peak voxels in the right sub-gyral and left superior temporal gyrus, whereas increasing Impulsive non-conformity was associated with decreased IPS in a cluster (k = 108) with peak voxels in bilateral precuneus and left middle temporal gyrus.

The negative relationship between the Imagination sub-scale of the AQ and Unusual Experiences sub-scale of the OLIFE was evident in different patterns of IPS (Supp Figure 4D), with increasing difficulties in imagination being associated with increased IPS in a cluster (k = 73) with two peak voxels in the right middle frontal gyrus, and higher Unusual Experiences associated with decreased IPS in a cluster (k = 100) with two peak voxels in the right precuneus.

Entering the AQ sub-scales of Attention Switching and Communication and the OLIFE sub-scale of Cognitive Disorganisation as regressors revealed an increased IPS associated with Attention Switching in two clusters (k = 166), the first of which with peak voxels in the right posterior insula and superior temporal gyrus, the second of which with peak voxels in the left superior temporal gyrus and transverse temporal gyrus (Supp Figure 4E).


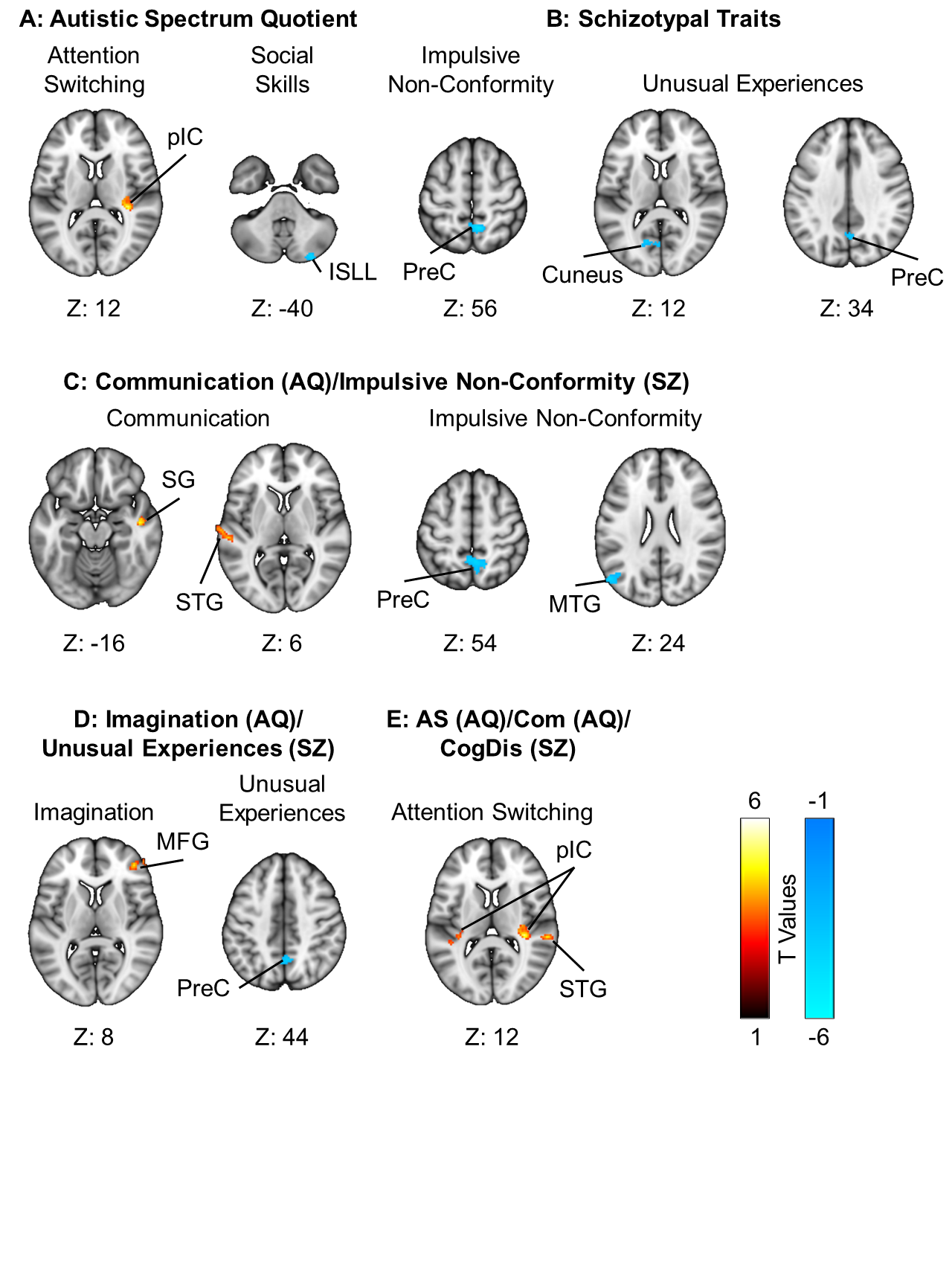


**Supplementary Figure 4.** Inter-subject phase synchronisation of neural activity is related to individual differences in autistic traits (A) and schizotypal traits (B), and can differentiate inter-trait relationships between Communication and Impulsive Non-Conformity (C), Imagination and Unusual Experience (D), and Attention Switching, Communication, and Cognitive Disorganisation (E). Abbreviations: pIC = posterior insula cortex, ISLL = inferior semi-lunar lobe, PreC = Precuneus, SG = sub-gyral, STG = superior temporal gyrus, MTG = middle temporal gyrus, MFG = middle frontal gyrus.

**Autistic and Schizotypal traits associated with neural activity in response to biological motion**

We next conducted a GLM analysis to establish the extent to which autistic and schizoptyal traits are associated with neural activity in response to biological motion. The first level analysis with biological motion as a regressor were entered into a second-level analysis with trait scores as a regressor.

*Intra-Trait analyses (p < 001, cluster corrected):* When all five sub-scales of the AQ were entered as regressors, the sub-scale of Imagination showed a negative relationship with the neural response to biological motion in a cluster (k = 157) with three peak voxels in the left lingual gyrus. When all four sub-scales of the OLIFE were entered, the sub-scale of Impulsive Non-Conformity showed a positive relationship with the neural response to biological motion in a cluster (k = 110) with a peak voxel in the left post-central gyrus.

*Inter-Trait analyses (p < 001, cluster corrected):* The OLIFE sub-scale of Impulsive Non-Conformity, with the AQ sub-scale of Communication as a covariate, was positively associated with the neural response to biological motion in a cluster (k = 111) with two peak voxels in the left post-central gyrus.


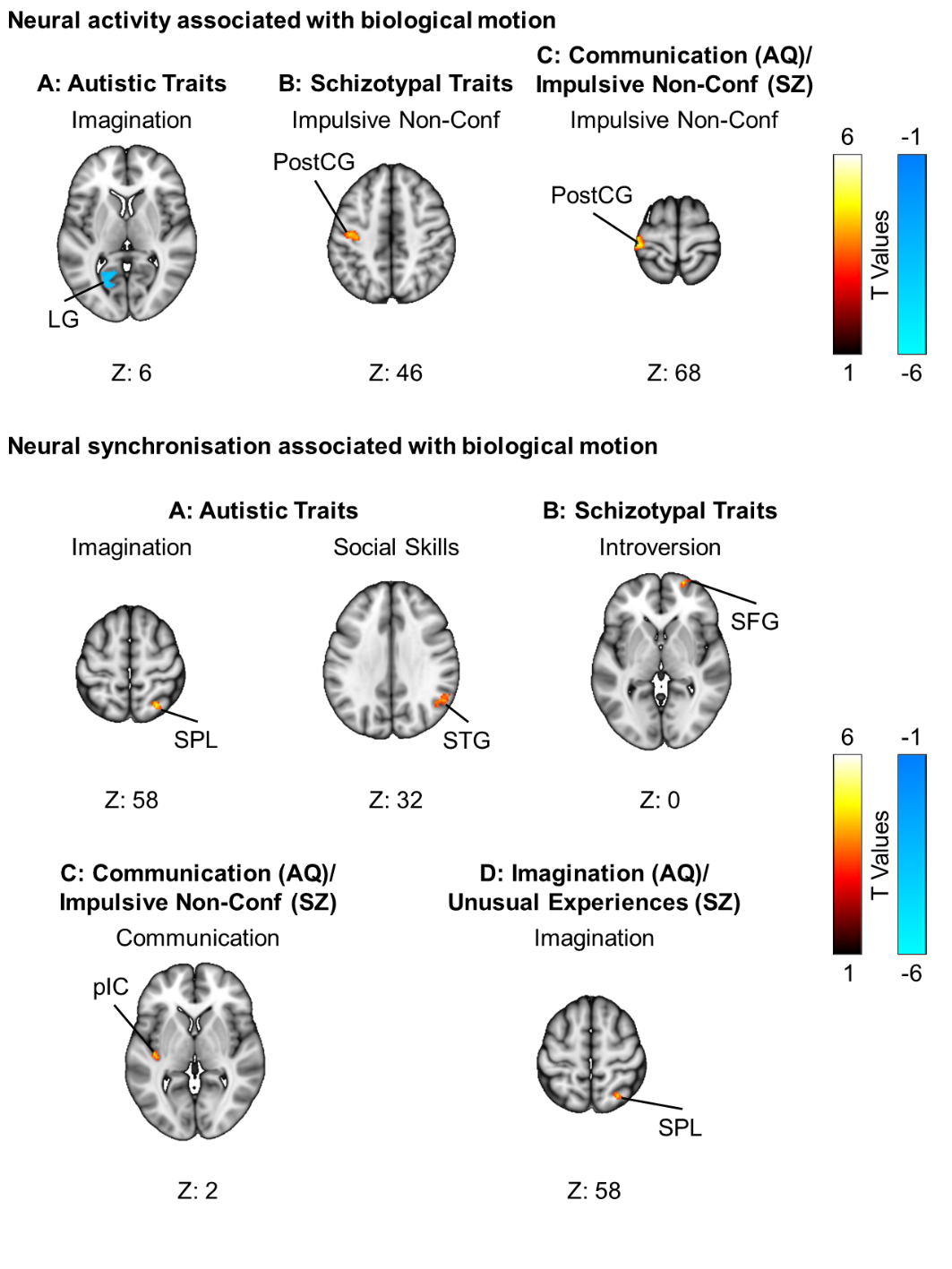


**Supplementary Figure 5.** The magnitude of neural activity associated with biological motion perception varies with individual differences in autistic traits of imagination when other autistic traits are controlled for (A), and the schizotypal trait of impulsive non-conformity when other schizotypal traits are controlled for (B) and when the correlated autistic trait of communication is controlled for (C). Abbreviations: LG = lingual gyrus, PostCG = post central gyrus

**Autistic and Schizotypal traits associated with neural synchronisation in response to biological motion**

For each individual the inter-subject phase synchronisation was correlated with biological motion for each voxel, and the r values were Fischer z transformed. These first-level results were entered into a second level analyses with trait scores as regressors.

*Intra-Trait analyses (p < 001, cluster corrected):* With all AQ sub-scales entered as regressors, the relationship between IPS and biological motion increased with Imagination scores in a cluster (k = 49) with a peak voxel in the right superior parietal lobe, and with Social Skills scores in a cluster (k = 71) with three peak voxels in the right superior and middle temporal gyri. With all OLIFE sub-scales entered as regressors, the relationship between IPS and biological motion increased with Introversion scores in a cluster (k = 70) with three peak voxels in the right superior and medial frontal gyri.

*Inter-Trait analysis (p < 001, cluster corrected):* The relationship between IPS and biological motion increased with scores on the AQ sub-scale of Communication, with the OLIFE sub-scale of Impulsive Non-Conformity as a covariate, in a cluster (k = 38) with a peak voxel in the left posterior insula cortex. The relationship between IPS and biological motion increased with the AQ sub-scale of Imagination, with the OLIFE sub-scale of Unusual Experiences as a covariate, in a cluster (k = 40) with a peak voxel in the right superior parietal lobe.


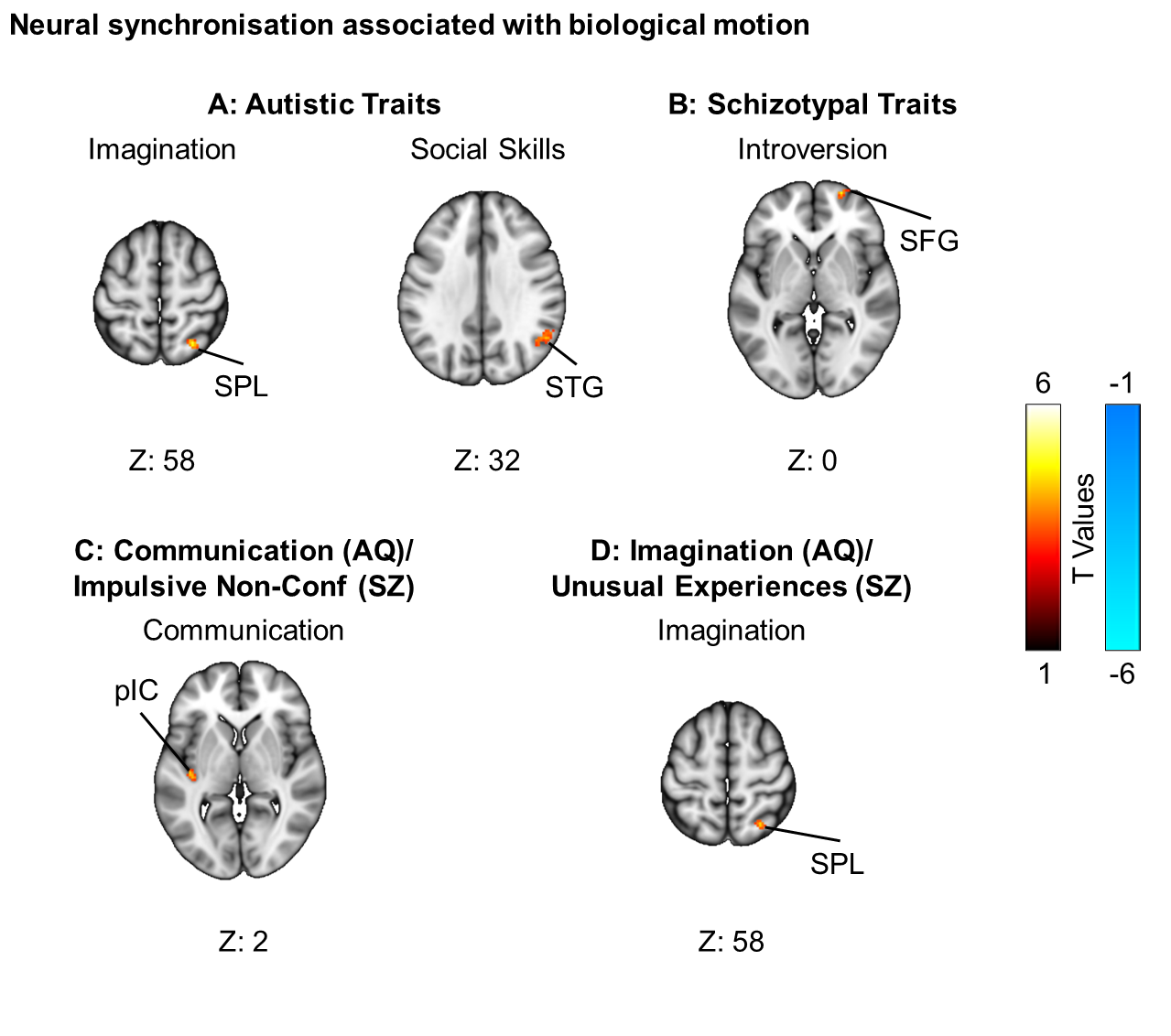


**Supplementary Figure 6.** Neural synchronisation associated with biological motion perception varies with individual differences in autistic traits and schizotypal traits. The autistic traits of imagination and social skills are associated with increased neural synchronisation when other autistic traits are controlled for (A), and the schizotypal trait of introversion is associated with increase neural synchronisation when other schizotypal traits are controlled for (B). Neural synchronisation increased with the autistic traits of communication (when the correlated schizotypal trait of impulsive non-conformity was controlled) (C), and imagination (when the correlated schizotypal trait of unusual experiences was controlled for) (D). Abbreviations: SPL = superior parietal lobe, STG = superior temporal gyrus, SFG = superior frontal gyrus, pIC = posteior insular cortex.
